# SDNET 2025- Annotated dataset of steel bolted connection evaluation

**DOI:** 10.1016/j.dib.2025.112254

**Published:** 2025-11-07

**Authors:** Faezeh Jafari, Sattar Dorafshan

**Affiliations:** a Department of Civil Engineering, University of North Dakota, Grand Forks, ND 58202, USA; b Advanced Transportation Infrastructure Center, University of North Dakota, Grand Forks, ND 58202, USA

**Keywords:** Structural defect networks, Defected steel connection, Bolt and nuts, Missing, loosen

## Abstract

Steel structures, particularly bridges and high mast poles, must be carefully inspected for their connection integrity, mmissing connection parts (missing or loose bolts and nuts), holes, or other defects negatively impact the structure’s ability to safely carry loads, potentially leading to catastrophic failures on transportation infrastructure. Traditional inspection methods are time-consuming, labour-intensive, unsafe, and costly—especially for tall structures. The use of Unmanned Aerial Systems (UAS) for visual imaging, combined with object detection, to identify defective bolts in steel structures has recently been implemented for structural condition assessment. However, there is a lack of publicly available datasets for this type of defect detection and model development, limiting adaptation of AI for autonomous bolted connection evaluation. This paper presents a dataset that includes multiple types of bolt and nut defects, such as missing bolts, loosening bolt.The dataset included 324 annotated images for loosen bolt, 200 images with missing bolt, and 302 images with fixed bolt. The dataset was compiled to meet the need for automated condition assessment of defective bolted connections in steel structures using deep learning techniques. Therefore, the authors aimed to gather as much data as possible to create a diverse set of 826 images, capturing various types of defects. To make the data more generalizable, it was collected through visual inspections of street structures from multiple cities in the world. Additionally, to increase the dataset size, laboratory data were created with bolts exhibiting various defects, and images were captured from these samples. All images in this dataset are annotated with bounding boxes and masks, making them suitable for any type of object detection algorithm.

Specifications Table

**Table:** 

Subject	Engineering & Materials science
Specific subject area	*Civil engineering*
Type of data	*Images, CSV, and* JavaScript Object Notation (*JSON) file*
Data collection	The dataset was collected using a smartphone. Images of defective bolts (missing and loosened) were captured from both laboratory setups and real-world steel structures. It includes over 800 original images, along with an annotation file corresponding to the image dataset.
Data source location	United State and Turkey
Data accessibility	Repository name: SDNET 2025- Defective Bolts and Nuts Steel dataset Data identification number: 10.17632/cxnj6cp4rb.1 Direct URL to data: https://data.mendeley.com/datasets/cxnj6cp4rb/1
Related research article	Jafari, F., & Dorafshan, S. (2025). Condition assessment of bolted connections in steel structures using deep learning. Innovative Infrastructure Solutions, 10(2), 65.

## 1 Value of the Data

• The dataset facilitates the development and validation of deep learning models for automated condition assessment, reducing reliance on manual inspections and contributing to the creation of smarter cities.
• The dataset aids in detecting defective bolts and nuts, helping to prevent infrastructure failures and ensuring public safety. It is particularly useful for monitoring the health of structures.
• The dataset is annotated to include the different levels of loosened and missing bolts and nuts. The dataset includes annotations using both masks and bounding boxes, allowing flexibility for automated condition assessment of bolted connections.
• It can serve as a benchmark dataset for comparative studies on different deep learning architectures and feature extraction techniques for detecting defective bolts in steel structures.
• With the development of synthetically generated data—such as Generative Adversarial Networks (GANs), Blender-based superimposition, and data augmentation techniques—the dataset can serve as a foundation for creating larger, more generic datasets to enhance AI model robustness and generalization. The dataset can be used as a balanced dataset for different types of defects through data augmentation.
• The dataset can be used by educators to teach applications of AI, ML, and DL.

## 2 Background

The increasing need for automated inspection in transportation infrastructure has highlighted the importance of AI-based methods to structural defect detection in an autonomous manner [1]. Structural defect networks SDNETs have been generated and used for concrete structures and bridges defect identification (SDNET2018 and SDNET2021) [2,3]. However, automated condition assessment of bolted connections in steel structures have been hindered due scarcity of annotated and realistic dataset for bolt and nut connections [1]. The lack of a dataset from real in-service structures with defective bolts and nuts posed a significant challenge in training deep learning models to differentiate between defective and non-defective bolts [1]. By offering real-world defect samples, this dataset can advance automated condition assessment, aiding in the efficient maintenance and safety evaluation of transportation infrastructure and monitoring structures in real time. Research on detecting defective bolts in street structures is limited. Most studies relied on data collected under controlled environments [[4], [5], [6]], limiting the use of AI models in field data. Moreover, the bolt size and shape were constant in previous datasets [4,5], or they did not adequately address defective bolts as two main types of defects in steel structures [6]. Deep learning is widely used for infrastructure preservation, such as condition assessment and defect detection, since hands-on inspections are often time-consuming, costly, and, in some cases, unsafe due to structural height and hard to access connections [1,6]. A major limitation of past studies is their reliance on laboratory samples for training and testing deep learning models to detect defective bolts and nuts. Object detection algorithms like Faster R-CNN (FRCNN) and Mask R-CNN (MRCNN) have been commonly used, yet their accuracy decreases when applied to real-world structures with various types of defective bolts or nuts. Identifying defective bolts [1] is crucial for transportation departments across the United States, as undetected issues can lead to sudden structural failure. Notably, as of this study, no publicly available dataset with annotations exists for defective bolts and nuts in steel street structures. Previous literature shows that authors typically create their own laboratory datasets or collect data from steel structures with the same bolt sizes, nut sizes, and structural shapes, developing AI models to detect defective bolts or nuts from non-defective structures [[2], [3], [4], [5], [6], [7]]. They report the models' performance based on one type of defective bolt [1], but these models often struggle to generalize to real-world conditions where bolt sizes, shapes, defect types, and structural shapes vary significantly [1]. Consequently, there is a need for a comprehensive dataset that includes annotated defective and non-defective bolts from different in-service structures to enhance model robustness and applicability in real-world inspections.

## 3 Data Description

**Fig. 1:**
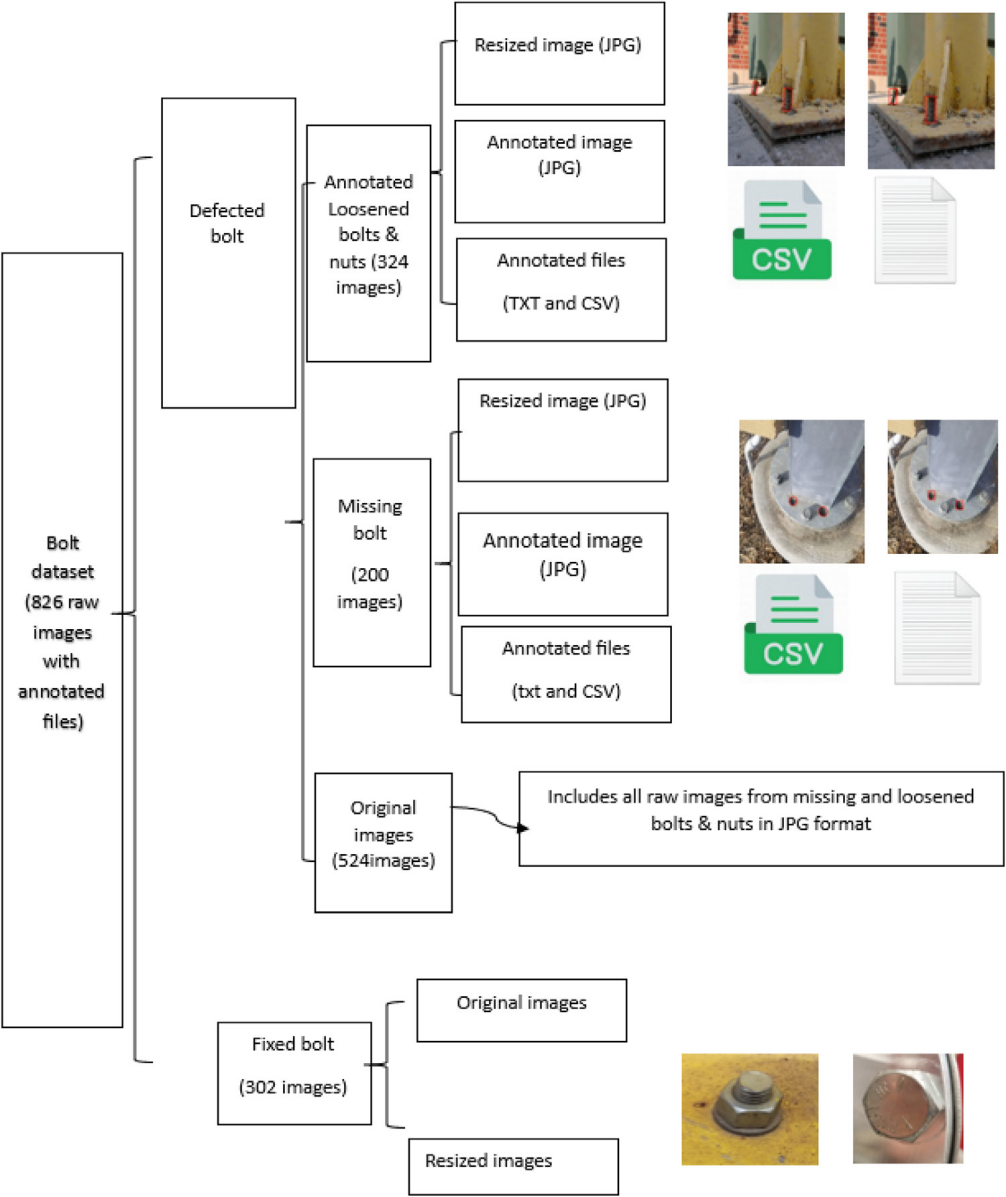
Dataset description.

The dataset in this study is categorized into two types of bolt-nut connections. Each folder includes a diverse set of defective bolts and nuts, varying in shape and size. Each folder contains annotated files in both bounding box and polygon annotation formats, along with the corresponding images. In this study, the images and associated annotation data were organized in a structured and systematic manner to ensure easy access and efficient processing. The data was divided into multiple categories based on the defect type observed in the images. As shown in Fig. 1, the folder structure is as follows

• First, the images were divided into two groups: the first group contains images of defective bolts, such as missing or loosened bolts, while the second group consists of images with no defects (fixed bolts).
• For fixed bolts, the original images and resized images (640 × 640) were placed in separate folders. The images were resized to ensure uniform dimensions for deep learning algorithms. For defective bolts, images with various defects, such as missing or loosened bolts and nuts, were organized in the original folder. All images were also annotated using polygons and bounding boxes in separate folders for loosened and missing bolts and nuts.
• The annotation was performed manually with the assistance of AI tools available in Roboflow [7] to ensure accurate image labelling. The author manually clicked around the defective bolts in the images, and the AI in Roboflow [7] highlighted the detected defects, prompting the annotator to confirm the defective areas. The annotator could then confirm or modify the identified areas. Using AI-assisted annotation in Roboflow facilitated dataset pre-processing, augmentation, and the export of annotations in multiple formats (CSV, JSON) for deep learning applications. Both bounding boxes and polygon segmentation were used, allowing detailed defect classification, such as missing and loosened bolts. This combination improved annotation efficiency and accuracy.
• After performing annotation, for the defective bolts, there are a total of 200 images with 449 defective bolts for missing bolts & nuts, and 324 images with 507 defective bolts for loosened bolts & nuts, resulting in a total of 524 images and 956 defective bolts across both categories.
• As shown in Fig. 1, for each type of defective bolt, the dataset includes resized images (640 × 640), annotated images using polygons, annotated images using bounding boxes, and annotation files in CSV and JSON formats.

## 4 Experimental Design, Materials and Methods

**Fig. 2:**
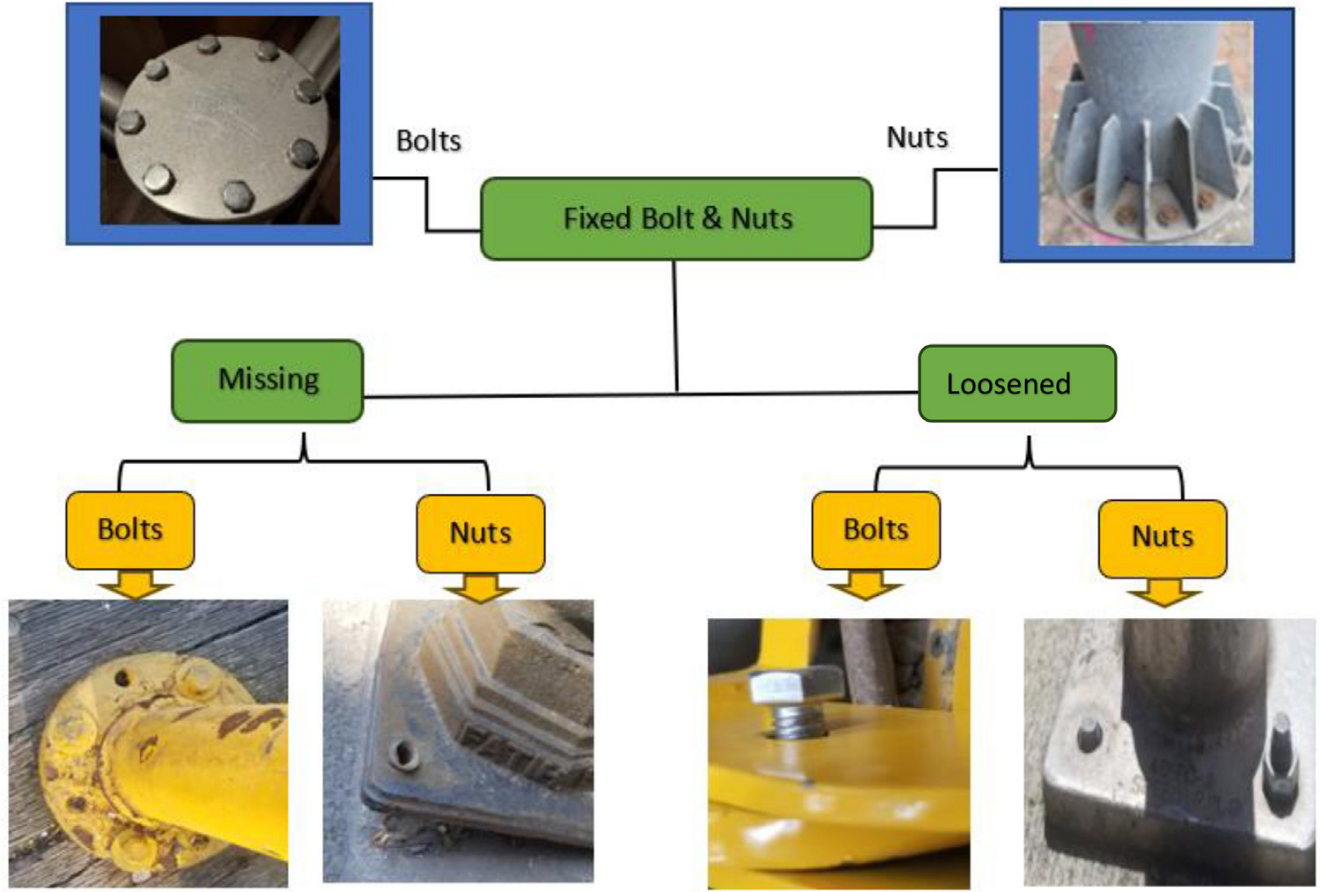
Different types of bolt and nut connections.

This dataset focuses on capturing data for defective bolts such as missing and loosened bolts in steel structures and annotated based on object detection algorithms. A dataset of 826 images, collected from laboratory setups and real-world structures in North Dakota, Baltimore, and Istanbul, was annotated for defect detection. In some states of United states, structures generally exhibit fewer defects, however, in cities like Baltimore, Istanbul, Arizona, and Seattle have a higher prevalence of deteriorated structures due to factors such as climate variations, exposure to moisture, and aging infrastructure [1]. Different locations were selected to ensure a diverse data set representing different environmental conditions and structural aging factors. The inclusion of multiple locations enhances the dataset’s robustness [1], allowing the deep learning model to generalize better across different structural conditions. Images were captured using a smartphone camera with a focal length of 8.604 mm, a resolution of 12 MP (4032 × 3024 pixels), an automatic shutter, and a CMOS sensor. The camera features optical image stabilization (OIS) and dual-pixel phase detection autofocus (PDAF). Images were taken at varying working distances ranging from 30 cm to 2 m under controlled lighting conditions. The lighting conditions varied, including artificial illumination in a laboratory setting as well as natural lighting in real-world environments. Based on Fig. 2**,** the images in dataset were categorized in the following subsets:

• Missing Bolt: A bolt or nut that is completely absent in the image, having entirely separated from structures. This defect typically occurs in structures subjected to significant cyclic loads or wind.
• Loosened Bolt: A bolt that is not fully tightened but remains connected to the steel structure, potentially posing a risk. This defect typically occurs due to structural loads, vehicle and human loads, or wind forces. Loosened bolts can have different degrees of loosening.
• Fixed Bolt: A properly secured bolt with no defects. This is a standard condition in structures without defects. Images of fixed bolts are included to provide data collection from both defective and non-defective conditions.

Multiple images from different angles of structures were captured with effort to present maximum defect visibility in dataset; finally, the best images with selected and represent in this dataset.

Table 1 presents a breakdown of the number of annotated images by defect type and location. The dataset includes images collected from both outdoor and indoor environments. This distribution highlights the geographical and structural diversity of the dataset, ensuring that the model is exposed to a wide range of real-world conditions and connection types. The dataset captured in different location, time of date, and camera angle. A dark environment can significantly reduce visibility, leading to failures in detecting small defective bolts in ancillary structures. In this dataset, images were captured randomly throughout the day (including before and after sunset), enabling any AI model to train bolt defect detection under varying lighting conditions. Furthermore, capturing bolts from diverse angles and directions contributed to a more robust dataset and resulted in higher testing accuracy. The inclusion of background variations in some images further enhanced dataset diversity, allowing the model to focus on identifying defective bolts within steel structures while disregarding irrelevant surrounding objects. As reported in Reference [1], the accuracy rate increased from 0.85 to 0.95 when the number of images with diverse parameters, such as angle and background, was increased [1].

**Table 1:** Number of annotated images by defect type and data source.

Location / connection’s type	Turkey (Field)	USA (Field)	UAS (Laboratory)
Loosen bolt	110	152	62
Missing bolt	64	67	69
Fixed bolt	50	232	20

**Fig. 3:**
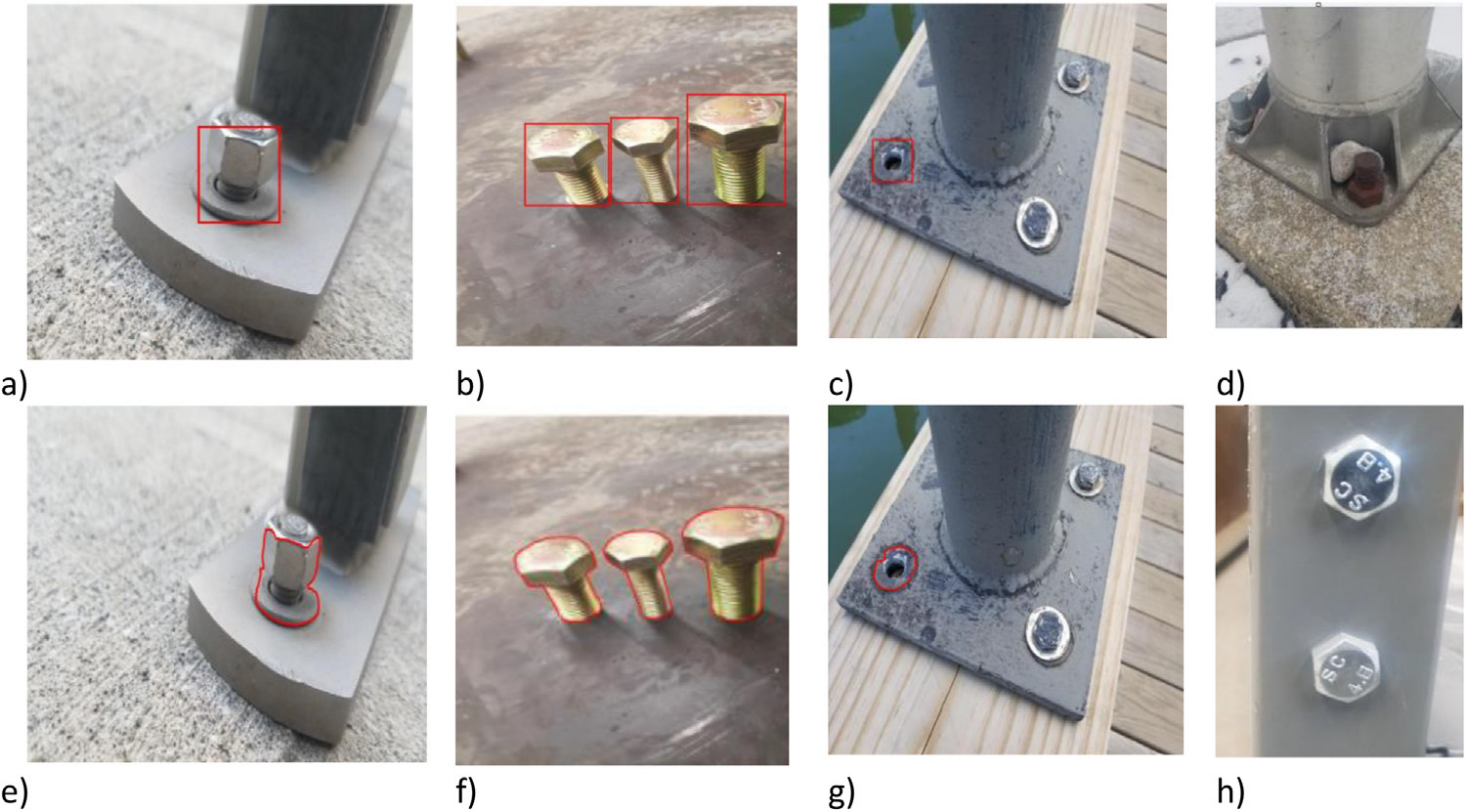
Annotated images with bounding box and mask annotations: (a & b) Loosened bolts with bounding box annotation; (c & g) Missing bolts with bounding box and mask annotations; (d & h) Fixed bolts; (e & f) Loosened bolts with mask annotation.

Fig. 3 shows images including defective bolt (missing and loosen bolt) and fixing one with bounding box and mask annotation. The dataset used in Ref. [1] was annotated and cross-checked by both authors, who possess expertise in civil engineering. To ensure consistency, the annotation process was iteratively refined, with particular attention given to defining defect boundaries and labelling categories. After each annotation round, FRCNN and MASKRCNN object detection models were trained, and K-fold cross-validation was performed, using nine subsets for training and one for testing. By repeatedly evaluating the AI model on diverse subsets and visually inspecting its outputs, inconsistencies in the annotations were identified and subsequently corrected. It was observed that inaccurate annotations led to a reduction in the detection accuracy of defective bolts compared to intact bolts [1]. Nevertheless, the results demonstrated that the accuracy rate exceeded 85 % across all datasets. After the annotations were done by experts, Roboflow was used for automatic annotations to compare the accuracy of AI-generated annotations with the manual annotations. The results were carefully reviewed, and any discrepancies were identified and corrected.

## Limitations

Inspector’s Judgment: The primary goal of this study is to create a dataset for this type of defect. The annotations provided in this paper are intended to assist readers in using the data for object detection and deep learning applications. The annotation of defective bolts is based on our judgment and may be re-annotated for other defect types, such as corrosion, or for different objectives. Any errors found in the annotations should not be considered a critique of the paper itself.

Limited Perspective: The images were not captured during formal hands-on inspections, nor were they taken by a UAS. As a result, the first-person view (FPV) represented in this dataset may differ from those typically obtained during field inspections. Geometric transformation techniques can be applied to adjust the FPV. Additionally, if available, images captured by a UAS would be valuable for training AI models.

Measurement of Objects: The size and geometry of defects were neither controlled nor measured, and these attributes were not reported. Due to the diversity of the dataset, it was not feasible to maintain consistent sizes for defective bolts and structures.

## Ethics Statement

This study does not involve human participants, animals, or sensitive data. The dataset was collected exclusively for research purposes using a personal mobile device, ensuring that there are no ethical concerns regarding privacy or confidentiality.

## CRediT Author Statement

**Faezeh Jafari:** Data Collection, Data Curation, Methodology, Writing – Original Draft, Data Annotation, **Sattar Dorafshan:** Supervision, Review & Final Draft, Organization.

## Data Availability

SDNET 2025- Annotated Dataset of Steel Bolted Connection Evaluation (Original data).
